# Supplementary material for: Age-dependent Pdgfrβ signaling drives adipocyte progenitor dysfunction to alter the beige adipogenic niche in male mice
Source: Nat Commun. 2023 Mar 31;14:1806. doi: 10.1038/s41467-023-37386-z (PMC10066302; doi:10.1038/s41467-023-37386-z)
Supplement: Supplementary file 1 — Supplementary Information [file 41467_2023_37386_MOESM1_ESM.pdf]

Supplementary Figure 1

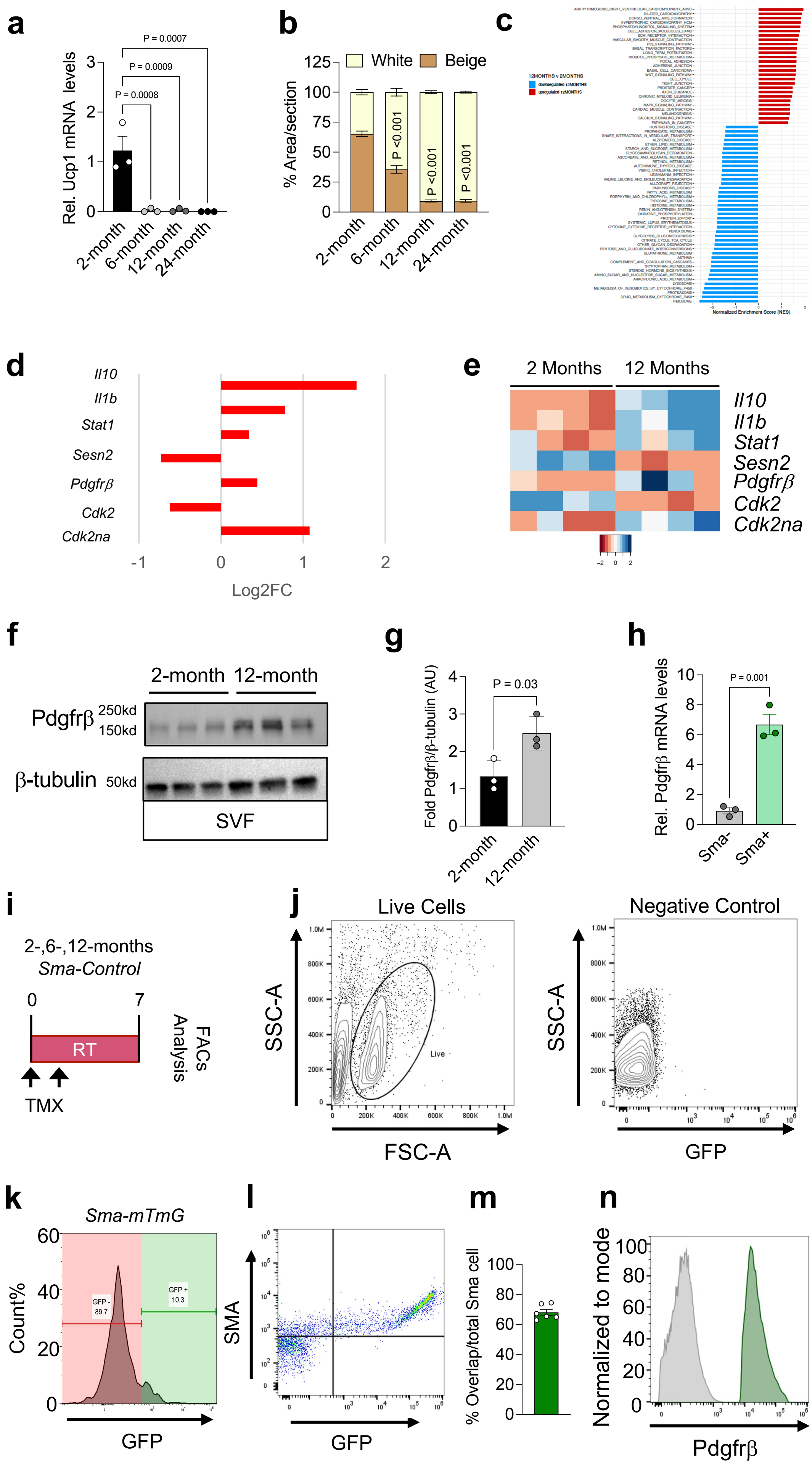

Supplementary Figure 1. Ageing promotes beige adipogenic failure and is associated with elevated levels of Pdgfrβ a, Ucp1 mRNA expression within dorsolumbar iWAT depots from two-, six-, twelve-, and twenty-four-month-old cold exposed (seven-days) male mice (n = 3 biologically independent mice/group examined over 2 experiments). **b,** Image quantification of Ucp1 immunofluorescence, identifying beige fat area within dorsolumbar iWAT sections from two-, six-, twelve-, and twenty-four-month-old cold exposed male mice (n = 3 biologically independent mice/group with 3 images per mouse quantified). **c-e,** Gene ontology and pathway analysis (**c**), gene enrichment (**d**), and heat map (**e**) of differential and senescence/ageing genes within the iWAT SVF of two- and twelve-month old male mice (*n* = 4 mice/group). **f,** Immunoblot of Pdgfrβ expression within the SVF of iWAT depots from two- and twelve-month-old male mice (*n* = 3 mice/group). **g,** Quantification of the immunoblot described in (**f**). **h,** Sma (-) and Sma (+) cells were FACS isolated from iWAT depots from *Sma-Cre^{ERT2}; R26^{mGFP}* mice. Pdgfrβ mRNA expression was assessed (*n* = 3 mice/group). **i,** Experimental schema: Sma-Control$^{(mGFP)}$ mice were administered one dose of tamoxifen for two consecutive days; subsequently, the SVF was isolated and Sma-mGFP+ cells were sort-purified. **j.** Representative flow cytometry plots demonstrating live cell and negative control gating strategies. **k, l,** Representative FACs histogram (**k**) and profile (**l**) of iWAT SV cells isolated from TMX pulsed Sma-Control mice. Similar plots were used to collect and identify Sma-mGFP+ cells under various conditions. **m,** Quantification of FACs analyzed Sma-mGFP colocalized with endogenous Sma over total antibody identified Sma expression within the SVF of iWAT depots from plots described in (**i**) (n = 6 biologically independent mice/group). **n,** Representative FACS histogram of Pdgfrβ expression within Sma+ beige APCs from two- (grey) and twelve-month (green) old male mice (*n* = 3 mice/group). Data are presented as mean values ± SEM. Data was analyzed by two-tailed Student's *t*-test. Source data are provided within the Source Data file. The full list of genes and normalized counts for the gene expression analysis can be found in Supplementary Data 1 and 2.

Supplementary Figure 2

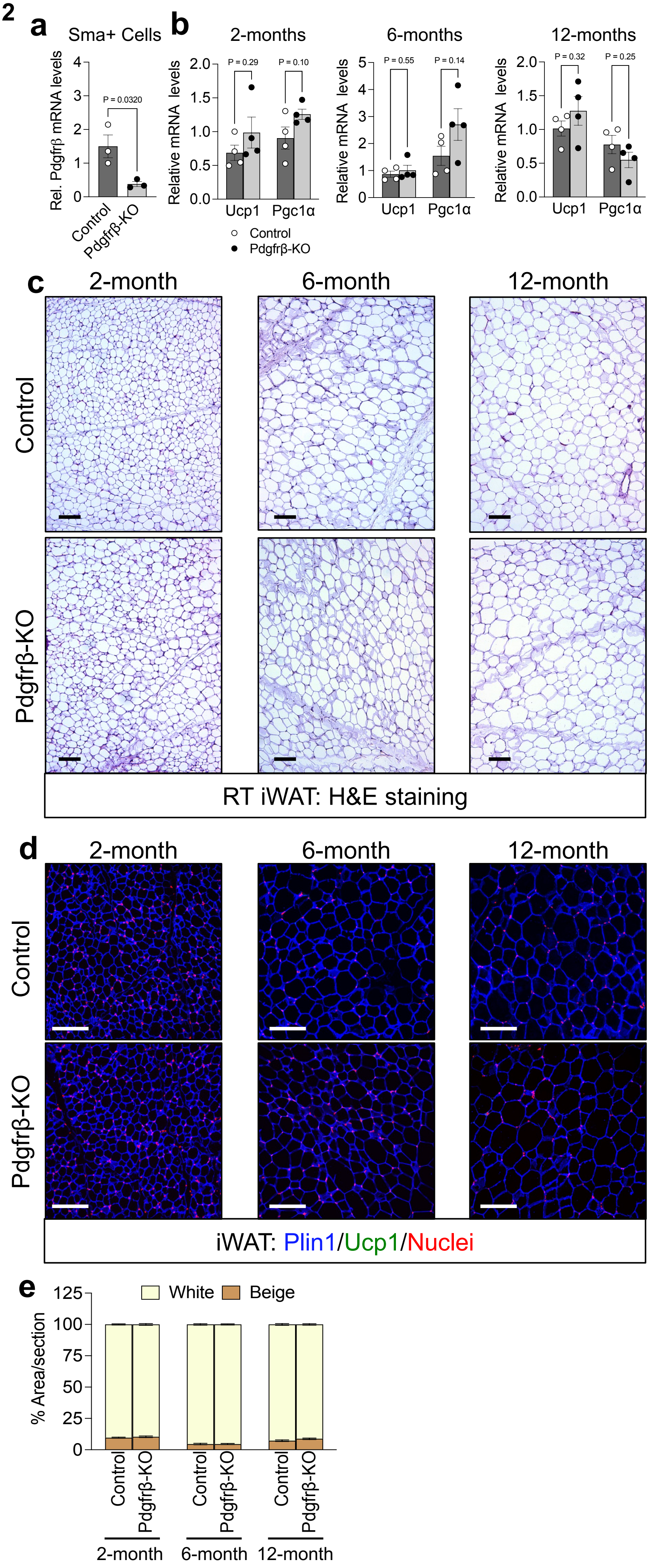

Supplementary Figure 2: Phenotypic analysis of room temperature Sma-Pdgfrβ-KO mice. a, Pdgfrβ mRNA expression within FACS isolated Sma+ cells from Sma-Control and Sma-Pdgfrβ-KO mice (n = 3 or 4 biologically independent mice/group examined over 2 experiments). **b,** Two-, six-, and twelve-month-old Sma-Control and Sma-Pdgfrβ-KO were administered one dose of TMX for two consecutive days. Dorsolumbar iWAT depots from mice maintained at RT were examined for Ucp1 and Pgc1α gene expression (n = 4 biologically independent mice/group examined over 2 experiments). **c,** Representative images of H&E staining of the dorsolumbar iWAT depot from mice described in (**b**) (x10 magnification, scale bar 100 µm). **d,** Representative images of Plin1 (blue) and Ucp1 (green) immunostaining of dorsolumbar iWAT depots from mice described in (**b**) (x20 magnification, scale bar 100 µm). **e,** Beige adipocyte quantification within dorsolumbar iWAT sections from RT maintained two-, six-, and twelve-month-old Sma-Control and Sma-Pdgfrβ-KO mice (*n* = 3 images/mouse; 3 mice/group) as described in (**b**). Data are presented as mean values ± SEM. Data was analyzed by two-tailed Student's *t*-test. Source data are provided within the Source Data file.

Supplementary Figure 3

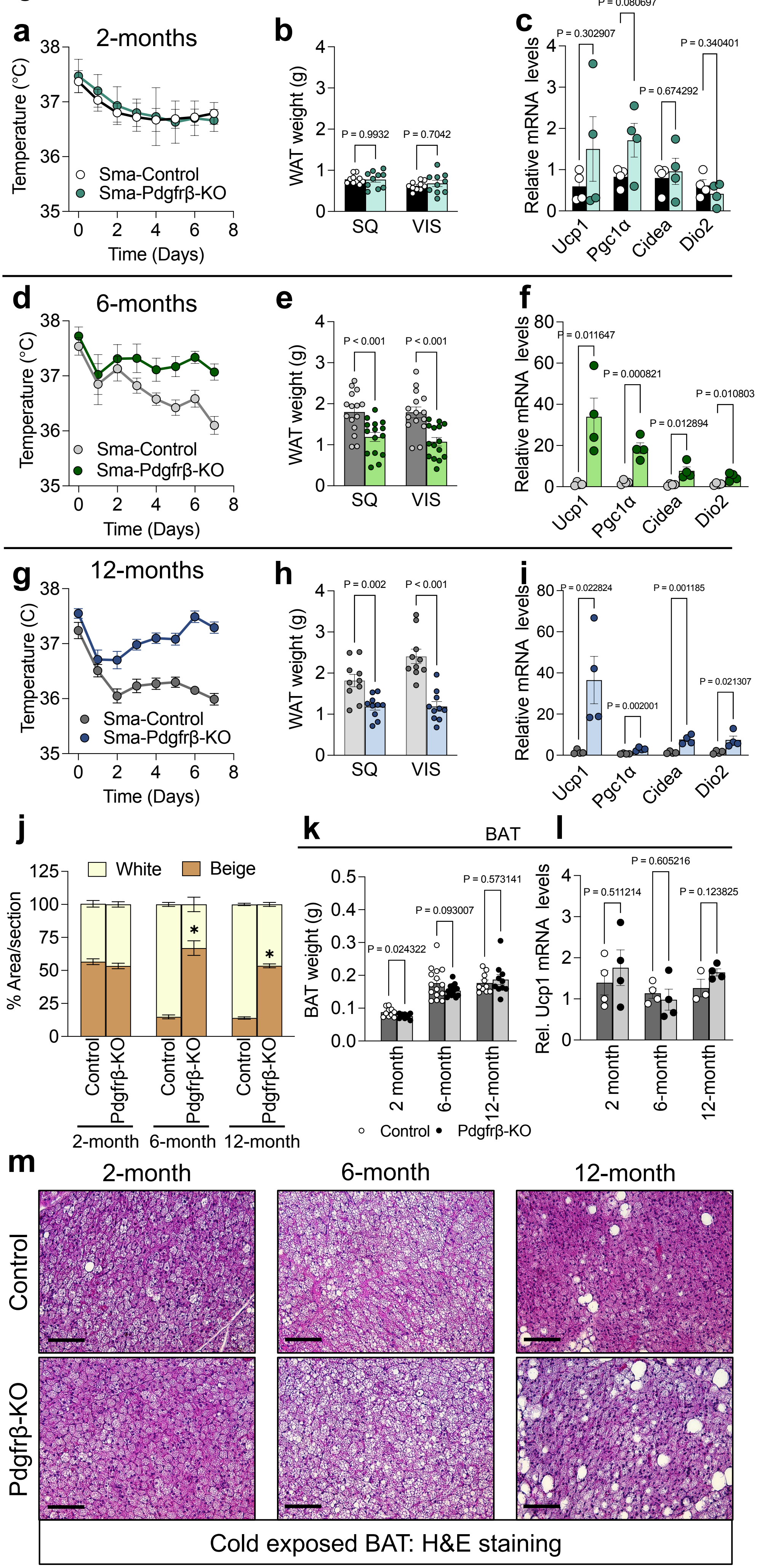

Supplementary Figure 3: Phenotypic analysis of cold-exposed Sma-Pdgfrβ-KO mice. a-c, Two-month-old Sma-Control and Sma-Pdgfrβ-KO male mice were administered TMX and randomized to cold exposure for seven days (*n* = 10 mice/group). Rectal temperature (**a**), WAT weight (**b**), and thermogenic gene expression (**c**) (*n* = 4 mice/group) were assessed. **d-f,** Six-month-old Sma-Control and Sma-Pdgfrβ-KO male mice were administered TMX and randomized to cold exposure for seven days (*n* = 15 mice/group). Rectal temperature (**d**), WAT weight (**e**), and thermogenic gene expression (**f**) (*n* = 4 mice/group) were assessed. **g-i,** Twelve-month-old Sma-Control and Sma-Pdgfrβ-KO male mice were administered TMX and randomized to cold exposure for seven days (*n* = 10 mice/group). Rectal temperature (**g**), WAT weight (**h**), and thermogenic gene expression (**i**) (*n* = 4 mice/group) were assessed. **j,** Beige adipocyte quantification within iWAT sections from cold exposed two-, six-, and twelve-month-old Sma-Control and Sma-Pdgfrβ-KO mice (*n* = 3 images/mouse; 3 mice/group)(*P = <0.001 mutants compared to age-match controls). **k, l,** BAT weight (**k**) and BAT Ucp1 mRNA expression (**l**) within cold exposed two-, six-, and twelve-month-old Sma-Control and Sma-Pdgfrβ-KO mice (n = 10 or 15 **(k)** or 4 **(l)** biologically independent mice/group). **m,** Representative images of H&E staining of BAT sections from cold exposed two-, six-, and twelve-month-old Sma-Control and Sma-Pdgfrβ-KO mice (x20 magnification, scale bars 100 µm) (Images representative of 3 independent experiments). Data are presented as mean values ± SEM. Data was analyzed by two-tailed Student's *t*-test. Source data are provided within the Source Data file.

Supplementary Figure 4

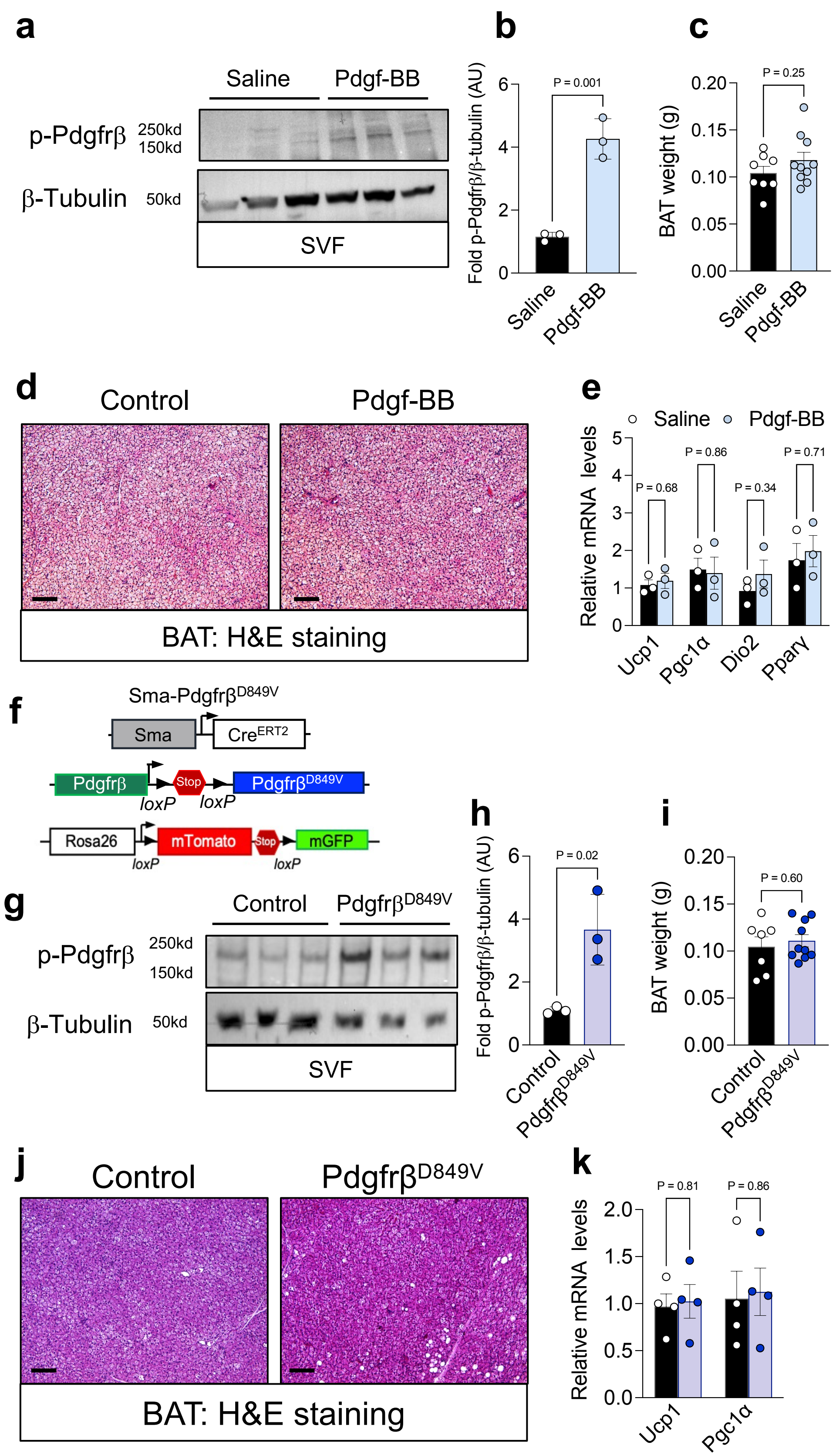

Supplementary Figure 4: Activating Pdgfrβ dampens beige fat development a, Two-month-old Sma-Control male mice were administered one dose of Saline (1X PBS 0.1% BSA) or Pdgf-BB (25 ng/mouse) for five consecutive days by IP injection at RT. Subsequently, the SVF was isolated from iWAT depots and immunoblotted against phosphorylated Pdgfrβ (p-Pdgfrβ) and β-tubulin levels (*n* = 3 mice/group). **b,** Quantification of immunoblot described in (**a**). **c,** BAT weight from mice described in (**a**) that were cold exposed for three-days (Vehicle *n* = 8; Pdgf-BB *n* = 10). **d,** Representative images of H&E staining of BAT sections from cold exposed mice described in (**c**) (x10 magnification, scale bar 100 µm). **e,** mRNA levels of denoted thermogenic genes within BAT from cold exposed mice described in (**c**) (*n* = 3 mice/group). **f,** Allelic combination used to generate Sma-Pdgfrβ^{D849V} mice. **g,** The SVF was isolated from iWAT depots from TMX-Induced Sma-Control and Sma-Pdgfrβ^{D849V} and immunoblotted against phosphorylated Pdgfrβ (p-Pdgfrβ) and β-tubulin levels (*n* = 3 mice/group). **h,** Quantification of the immunoblot described in (**g**). **i-k,** Two-month-old TMX-induced Sma-Control and Sma-Pdgfrβ^{D849V} male mice were cold exposed for seven days (Control *n* = 7; mutant *n* = 10). BAT weight (**i**), representative histology (**j**) (x10 magnification, scale bars 100 µm), and brown adipocyte gene expression (**k**) were evaluated. Data are presented as mean values ± SEM. Data was analyzed by two-tailed Student's *t*-test. Source data are provided within the Source Data file.

Supplementary Figure 5

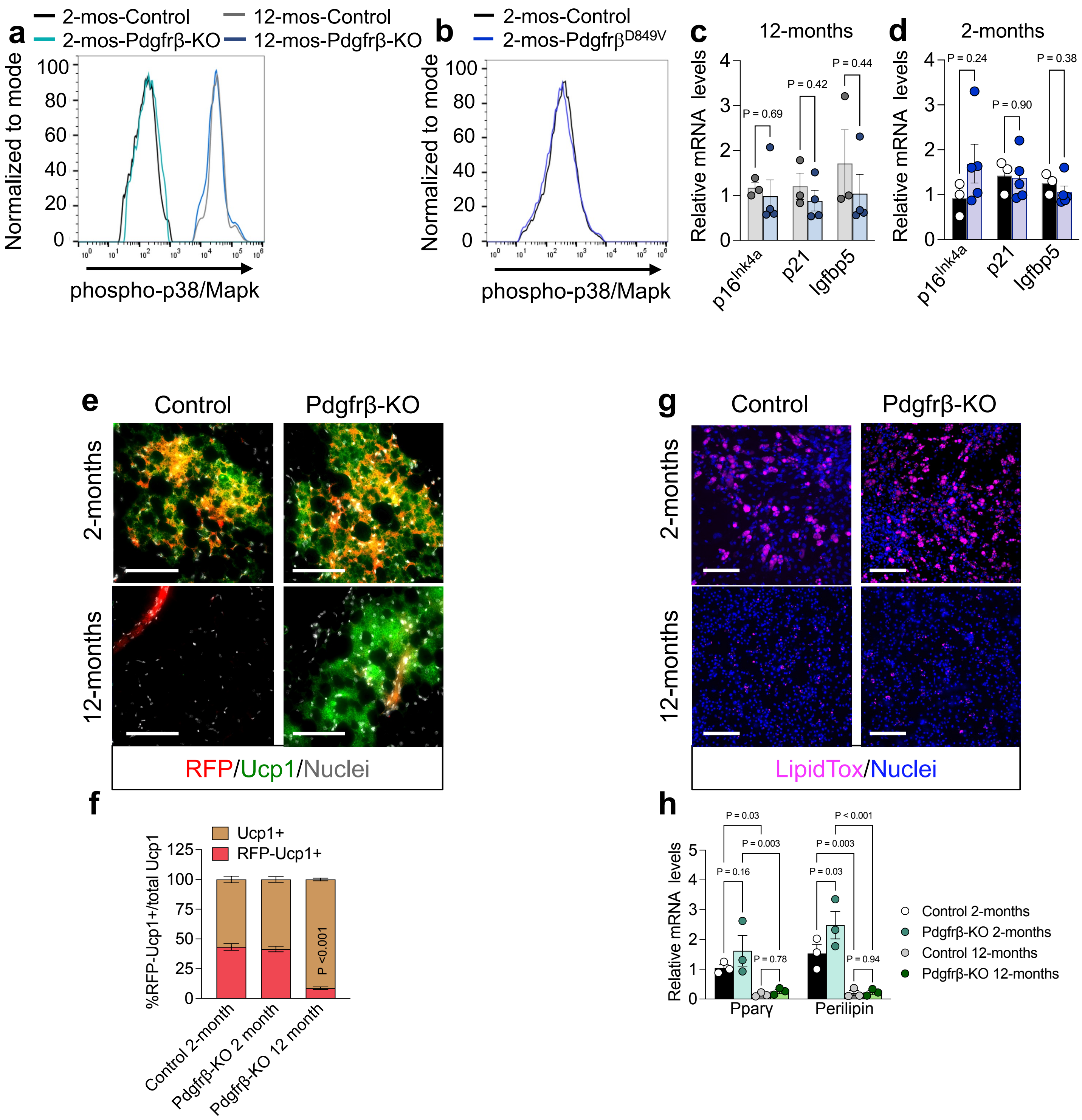

Supplementary Figure 5: Pdgfrβ activity does not alter senescence to induce beige fat development. **a,** Sma-^{mGFP}+ cells were FACS isolated from two- and twelve-month-old TMX-induced Sma-Control and Sma-Pdgfrβ-KO mice and examined for phosphorylated p38/Mapk by flow cytometry. **b,** Sma-^{mGFP}+ cells were FACS isolated from two-month-old TMX-induced Sma-Control and Sma-Pdgfrβ^{D849V} mice and examined for phosphorylated p38/MAPK by flow cytometry. **c,** mRNA levels of denoted senescence inducing genes within iWAT depots from twelve-month-old Sma-Control and Sma-Pdgfrβ-KO male mice (*n* = 3 or 4 mice/group). **d,** mRNA levels of denoted senescence inducing genes within iWAT depots from two-month-old Sma-Control and Sma-Pdgfrβ^{D849V} mice (Control *n* = 3; Pdgfrβ^{D849V} *n* = 5). **e,** Representative images of RFP (Sma-driven) and Ucp1 immunostaining and fate mapping analysis of iWAT sections from two- and twelve-month-old TMX-induced Sma-Control (R26^{RFP}) and Sma-Pdgfrβ-KO (R26^{RFP}) mice (x40 magnification, scale bars 100 μm). **f,** Quantification of RFP-Ucp1 overlap within sections described in (**e**) (*n* = 3 biologically independent mice/group with 3 images per mouse quantified). **g,** Representative images of lipid staining (LipidTox) of in vitro derived beige adipocytes from in vivo TMX-induced two- and twelve-month-old Sma-Control and Sma-Pdgfrβ-KO mice (x20 magnification, scale bars 100 μm). **h,** mRNA expression of adipocyte markers from cultures described in (i) (*n* = 3 mice/group). Data are presented as mean values ± SEM. Data was analyzed by two-tailed Student's t-test or two-way ANOVA multiple comparison. Source data are provided within the Source Data file.

Supplementary Figure 6

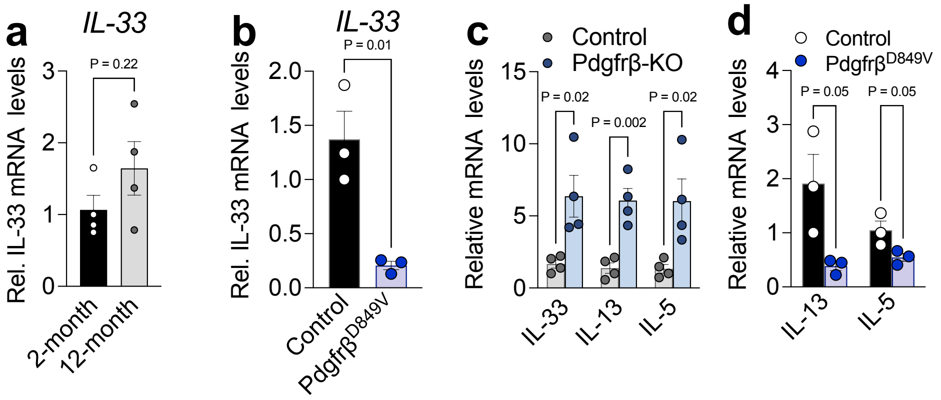

Supplementary Figure 6: Pdgfrβ regulates IL-33 to promote beige fat development a, mRNA expression of IL-33 within iWAT depots of two- and twelve-month-old Sma-Control mice maintained at RT (n = 4 biologically independent mice/group examined over 2 experiments). **b,** mRNA expression of IL-33 within iWAT depots of TMX-induced two-month-old Sma-Control and Sma-Pdgfrβ^{D849V} mice maintained at RT (n = 3 biologically independent mice/group examined over 2 experiments). **c,** mRNA expression levels of denoted type 2 cytokine immune markers from twelve-month-old Sma-Control and Sma-Pdgfrβ-KO mice (n = 4 biologically independent mice/group examined over 2 experiments). **d,** mRNA expression levels of denoted type 2 cytokine immune markers from two-month-old Sma-Control and Sma-Pdgfrβ^{D849V} mice cold exposed for two days (n = 3 biologically independent mice/group examined over 2 experiments). Data are presented as mean values ± SEM. Data was analyzed by two-tailed Student's *t*-test. Source data are provided within the Source Data file.

Supplementary Figure 7

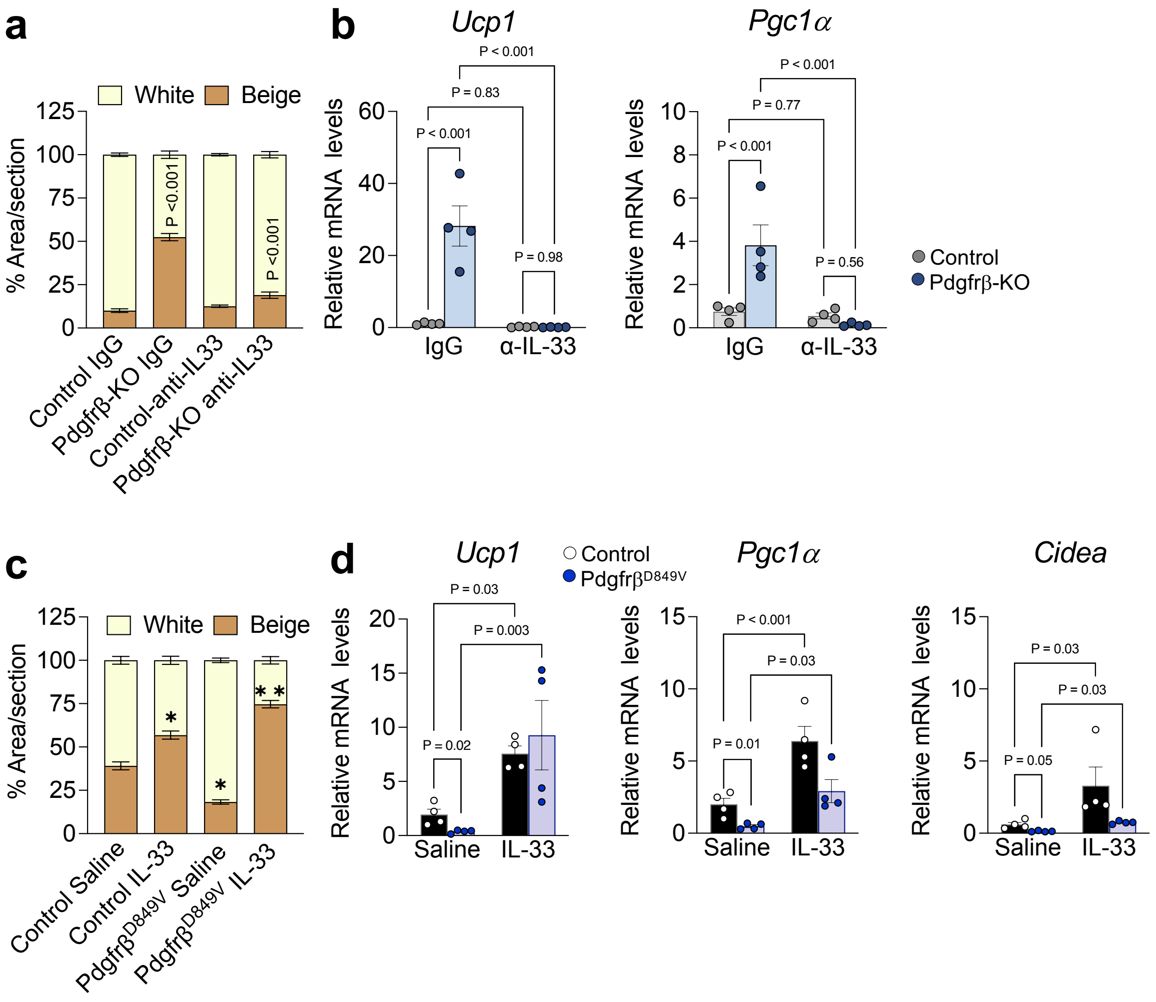

Supplementary Figure 7: IL-33 mediates Pdgfrβ-KO induced beige fat formation. a, Quantification of beige and white adipocyte area per section (*n*=3 images/mouse; 3 mice/group) from Ucp1 immunostained images (Fig. 5d) from twelve-month-old Sma-Control and Sma-Pdgfrβ-KO mice treated with IgG (1 µg/mouse) or anti-IL-33 (1 µg/mouse) antibodies. **b**, mRNA expression level of Ucp1 and Pgc1a from mice described in (**a**) (*n*=4 mice/group). **c,** Quantification of beige and white adipocyte area per section (*n*=3 images/mouse; 3 mice/group) from Ucp1 immunostained images (Fig. 5h) from two-month-old TMX-induced Sma-Control and Sma-Pdgfrβ^{D849V} were administered one dose of vehicle (0.1%BSA in 1xPBS) or recombinant murine IL-33 (12 µg/Kg) for five consecutive days and subsequently cold exposed for seven days (*P = < 0.001 compared to saline beige area; **P = < 0.001 Sma-Pdgfrβ^{D849V} compared to Sma-Pdgfrβ^{D849V} IL-33 treated beige area) **d**, mRNA expression level of Ucp1, Pgc1a, and Cidea from mice described in (**c**) (*n*=4 mice/group). Data are presented as mean values ± SEM. Data was analyzed by two-tailed Student's *t*-test or two-way ANOVA multiple comparison. Source data are provided within the Source Data file.

Supplementary Figure 8

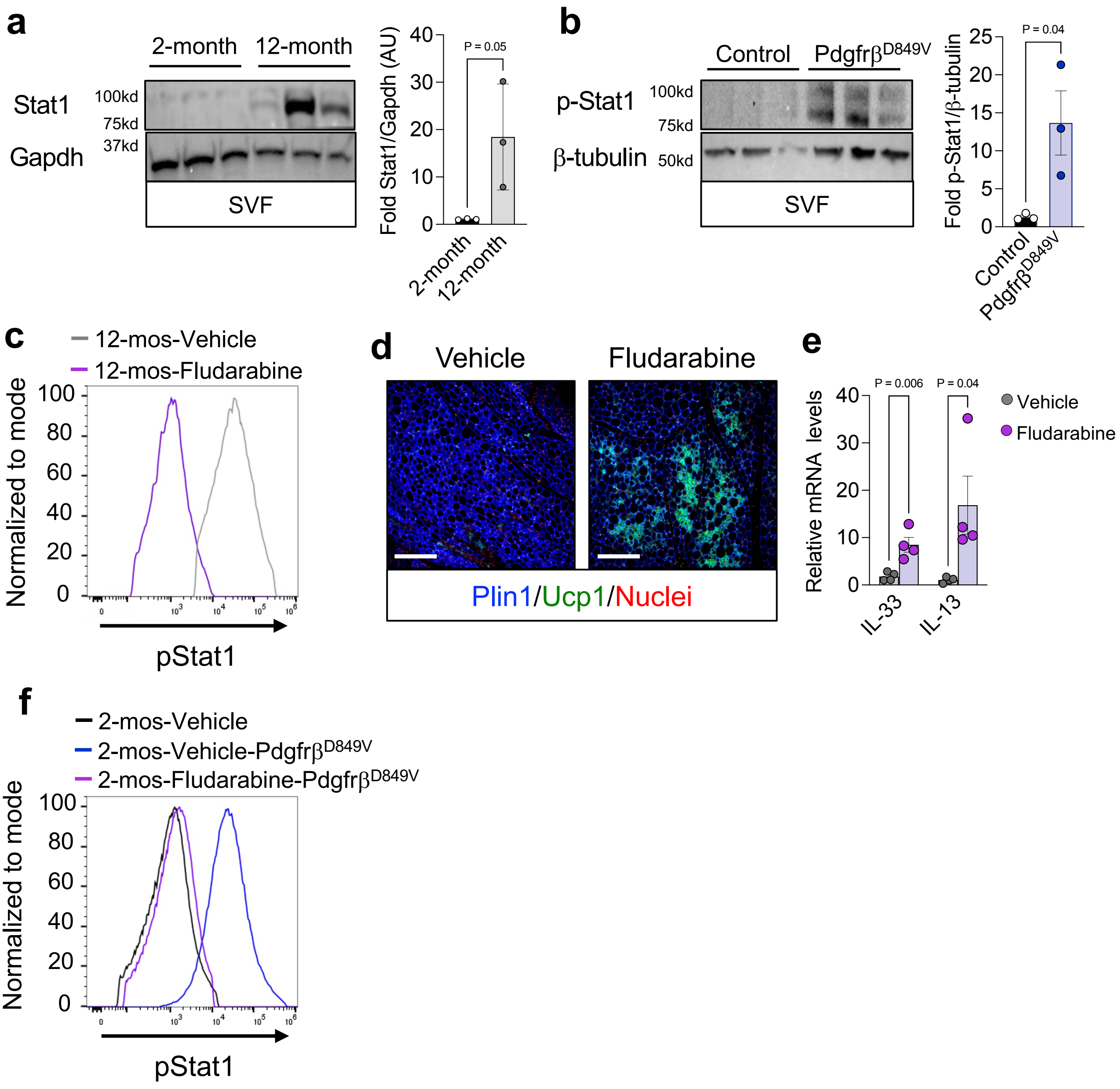

Supplementary Figure 8: Fludarabine restores beige adipogenic potential in ageing and Sma-Pdgfrβ^{D849V} mice. a, The SVF was isolated from iWAT depots from two- and twelve-month-old male mice and immunoblotted against total Stat1 and Gapdh was used as a loading control (*n*= 3 mice/group). Right: quantification of immunoblot. **b,** The SVF was isolated from iWAT depots from TMX-Induced Sma-Control and Sma-Pdgfrβ^{D849V} and immunoblotted against phosphorylated Stat1 (p- Stat1) and β-tubulin levels (*n*= 3 mice/group). Right: quantification of immunoblot. **c,** Representative flow cytometric histogram of phosphorylated Stat1 status within FACS isolated Sma-$^{mGFP+}$ cells from aged Sma-Control mice administered one dose of vehicle or fludarabine (3 mg/kg) for five consecutive days maintained at RT. **d,** Twelve-month-old TMX-induced Sma-Control mice were administered one dose of vehicle (5% DMSO) or fludarabine (3 mg/Kg) for five consecutive days and subsequently cold challenged for seven days. Representative images of Plin1 (blue) and Ucp1 (green) immunostaining of dorsolumbar iWAT sections (x20 magnification, scale bars 100 µm) (Images representative of 2 independent experiments). **e,** mRNA levels of IL-33 and IL-13 from mice described in (**d**) but after two of cold exposure (n = 4 biologically independent mice/group examined over 2 experiments). **f,** Representative flow cytometric histogram of phosphorylated Stat1 status within FACS isolated Sma-$^{mGFP+}$ cells from two-month-old Sma-Control and Sma-Pdgfrβ^{D849V} male mice administered one dose of vehicle or fludarabine (3 mg/kg) for five consecutive days maintained at RT. Western blots were performed using three independent mice. Data are presented as mean values ± SEM. Data was analyzed by two-tailed Student's *t*-test. Source data are provided within the Source Data file.

Supplementary Figure 9

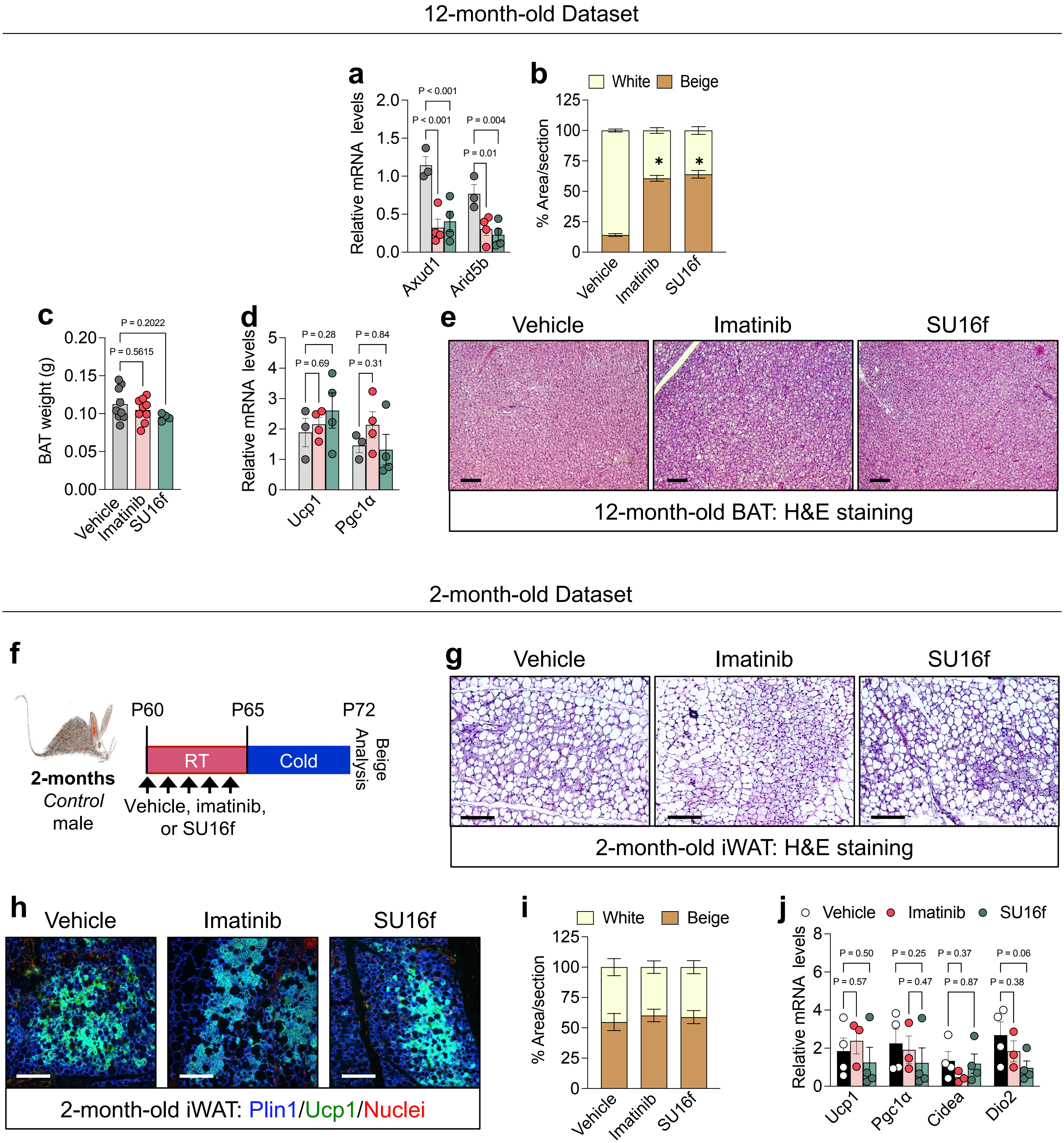

Supplementary Figure 9: Blocking Pdgfrβ restores beige adipogenic potential in ageing mice. a, Twelve-month-old male mice were administered one dose of vehicle (5% DMSO), imatinib (5 mg/Kg), or SU16f (2 mg/Kg) for five consecutive days and mRNA levels of Pdgfrβ genes in iWAT were assessed (*n*= 3 or 4 mice/group). **b,** Quantification of beige and white adipocyte area per section (*n*=3 images/mouse; 3 mice/group) from immunostained images in Fig. 7d (*P = <0.001 treated compared to vehicle). **c-e,** Mice described in (**a**) were cold challenged for seven days and BAT weight (**c**) (SU16f *n* = 4; Vehicle or imatinib n = 9 mice/group), BAT gene expression (**d**) (*n*= 3 or 4 mice/group) , and morphology (representative images of H&E staining x10 magnification, scale bar 100 μm) (**e**) was assessed. **f,** Experimental schema: two-month-old Sma-Control mice administered vehicle (5% DMSO), imatinib (5 mg/Kg), or SU16f (2 mg/Kg) for five consecutive days; subsequently mice were cold challenged for seven days (*n* = 4-13 mice/group). **g,** Representative images of H&E staining of dorsolumbar iWAT sections from mice described in (**f**) (x20 magnification, scale bars 100 μm). **h,** Representative images of Plin1 (blue) and Ucp1 (green) immunostaining of dorsolumbar iWAT sections from mice described (**f**) (x20 magnification, scale bars 100 μm). **i,** Quantification of beige and white adipocyte area per section (*n* = 3 images/mouse; 3 mice/group) from immunostained images in (**h**). **j,** mRNA levels of denoted thermogenic gene expression within iWAT depots from mice described in (**f**) (n = 3 or 4 biologically independent mice/group examined over 2 experiments). Data are presented as mean values ± SEM. Data was analyzed by two-tailed Student's *t*-test. Source data are provided within the Source Data file.

Supplementary Figure 10

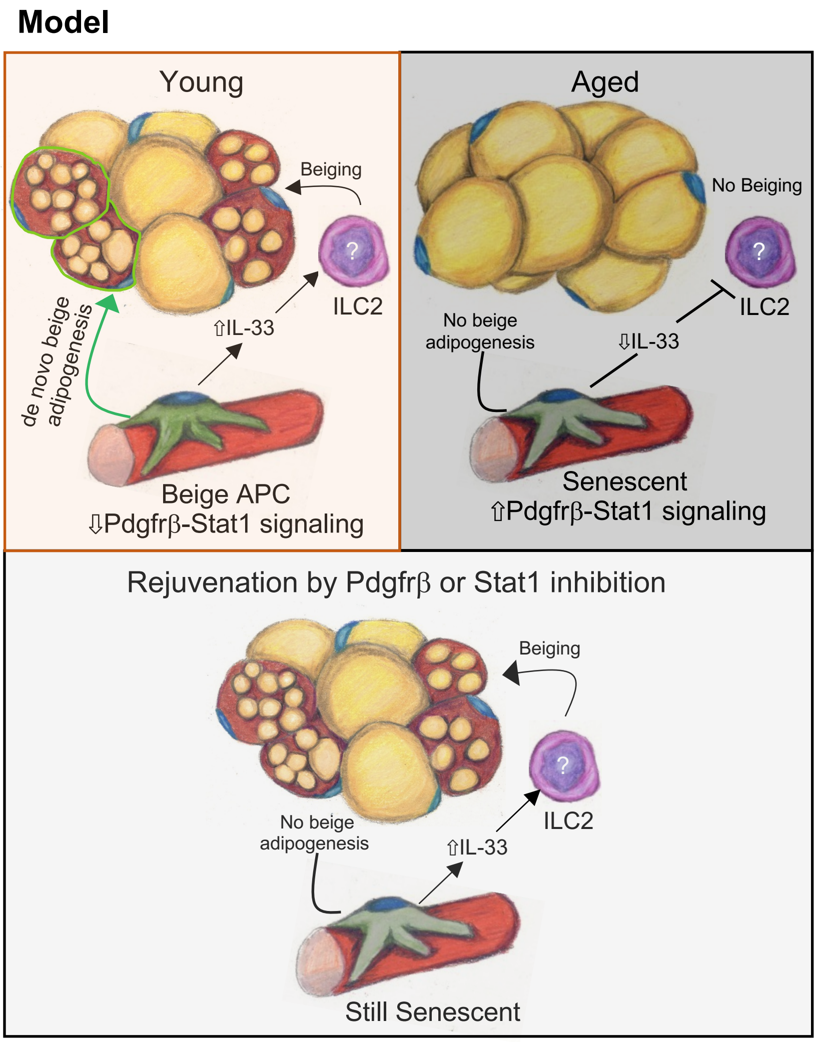

Supplementary Figure 10: Proposed Model. In the juvenile state, cold temperatures induce Sma marked beige APCs to generate beige adipocytes. Additionally, beige APCs further communicate with WAT-resident immunological niche cells to foster a beige adipogenic niche via IL-33 secretion. In the adult state, beige APCs appear to become senescent and are unable to generate beige adipocytes in response to cold temperature exposure. Accompanying cellular senescence, beige APCs acquire hyperactivated Pdgfrβ signaling which suppresses IL-33 availability via Stat1 phosphorylation. The concomitant loss of IL-33 blunts the immunological niche such as ILC2 or Th2-cells, dampening beige adipogenic responses. However, pharmacological blocking Pdgfrβ signaling restores beige adipogenic potential by elevating IL-33 gene suppression, stimulating the beige adipocyte development.

Supplementary Table 1

Gene	Forward	Reverse
Arid5b	GCTGATAACTTTACCGTCACAGT	GTGATGAGTTCGCGCCAAATC
Axud1	GTCTGTCCTCGGCTGTTGGAACC	CCACCTCAGCATCTCCAGCTTC
Cidea	TCTGCAATCCCATGAATGTC	CAGTGATTTAAGAGACGCGG
Dio2	ACACTGGAATTGGGAGCATC	ATGCTGACCTCAGAAGGGCT
Elovl3	TTCTCACGCGGGTTAAAAATGG	GAGCAACAGATAGACGACCAC
Fasn	GGAGGTGGTGATAGCCGGTAT	TGGGTAATCCATAGAGCCCAG
Igfbp5	CGCGGGGTTTGCCTCAACGA	CTGCGGCAGGGGCCTTGTTC
IL-5	CTCTGTTGACAAGCAATGAGACG	TCTTCAGTATGTCTAGCCCCTG
IL-13	CCTGGCTCTTGCTTGCCTT	GGTCTTGTGTGATGTTGCTCA
IL-33	TCCAACTCCAAGATTTCCCCG	CATGCAGTAGACATGGCAGAA
p16INK4a	CGCAGGTTCTTGGTCACTGT	TGTTCACGAAAGCCAGAGCG
p21	CGAGAACGGTGGAACTTTGAC	CAGGGCTCAGGTAGACCTTG
Perilipin	GGGACCTGTGAGTGCTTCC	GTATTGAAGAGCCGGGATCTTTT
hPdgfrb	AGCACCTTCGTTCTGACCTG	TATTCTCCCGTGTCTAGCCCA
mPdgfrb	AGGGGGCGTGATGACTAGG	TTCCAGGAGTGATACCAGCTT
Pgc1α	TATGGAGTGACATAGAGTGTGCT	CCACTTCAATCCACCCAGAAAG
Pparγ	TCGCTGATGCACTGCCTATG	GAGAGGTCCACAGAGCTGATT
Prdm16	ACACGCCAGTTCTCCAACCTGT	TGCTTGTTGAGGGAGGAGGTA
Ucp1	CGACTCAGTCCAAGAGTACTTCTCTT	GCCGGCTGAGATCTTGTTTC
Rn18S	GTAACCCGTTGAACCCCATT	CCATCCAATCGGTAGTAGCG

Supplementary Table 1. Primer sequences for qPCR gene expression analysis.
