# Supplementary material for: Age-dependent Pdgfrβ signaling drives adipocyte progenitor dysfunction to alter the beige adipogenic niche in male mice
Source: Nat Commun. 2023 Mar 31;14:1806. doi: 10.1038/s41467-023-37386-z (PMC10066302; doi:10.1038/s41467-023-37386-z)
Supplement: Supplementary file 3 — Description of Additional Supplementary Files [file 41467_2023_37386_MOESM3_ESM.pdf]

Description of Additional Supplementary Files

Supplementary Data 1: Bulk mRNA-seq analysis of 2 vs. 12-month-old SVF

Supplementary Data 2: GSEA Results: Gene sets enriched in 12 month old SVF

Supplementary Data 3: Bulk mRNA-seq analysis of 12 month cont vs. 12 month old PB KO SVF
