# Supplementary material for: Age-dependent Pdgfrβ signaling drives adipocyte progenitor dysfunction to alter the beige adipogenic niche in male mice
Source: Nat Commun. 2023 Mar 31;14:1806. doi: 10.1038/s41467-023-37386-z (PMC10066302; doi:10.1038/s41467-023-37386-z)
Supplement: Supplementary file 5 — Reporting Summary [file 41467_2023_37386_MOESM5_ESM.pdf]

nature portfolio

Corresponding author(s): Daniel Berry

Last updated by author(s): Feb 26, 2023

Reporting Summary

Nature Portfolio wishes to improve the reproducibility of the work that we publish. This form provides structure for consistency and transparency in reporting. For further information on Nature Portfolio policies, see our Editorial Policies and the Editorial Policy Checklist.

Statistics

For all statistical analyses, confirm that the following items are present in the figure legend, table legend, main text, or Methods section.

n/a	Confirmed	
☐	☒	The exact sample size (*n*) for each experimental group/condition, given as a discrete number and unit of measurement
☐	☒	A statement on whether measurements were taken from distinct samples or whether the same sample was measured repeatedly
☐	☒	The statistical test(s) used AND whether they are one- or two-sided<br>*Only common tests should be described solely by name; describe more complex techniques in the Methods section.*
☐	☒	A description of all covariates tested
☒	☐	A description of any assumptions or corrections, such as tests of normality and adjustment for multiple comparisons
☐	☒	A full description of the statistical parameters including central tendency (e.g. means) or other basic estimates (e.g. regression coefficient) AND variation (e.g. standard deviation) or associated estimates of uncertainty (e.g. confidence intervals)
☐	☒	For null hypothesis testing, the test statistic (e.g. *F*, *t*, *r*) with confidence intervals, effect sizes, degrees of freedom and *P* value noted<br>*Give P values as exact values whenever suitable.*
☒	☐	For Bayesian analysis, information on the choice of priors and Markov chain Monte Carlo settings
☒	☐	For hierarchical and complex designs, identification of the appropriate level for tests and full reporting of outcomes
☒	☐	Estimates of effect sizes (e.g. Cohen's *d*, Pearson's *r*), indicating how they were calculated

Our web collection on statistics for biologists contains articles on many of the points above.

Software and code

Policy information about availability of computer code

Data collection	Excel, Leica Application suite X, FlowJo version 10.8.1 , QuantStudio, BD FACSDiva Software version 9.4, Attune Cytometric Software 5.3.2415.0
Data analysis	Excel, Prism Graphpad7-9, FlowJo version 10.8.1, NIH-ImageJ software version 2.9.0/1.53t, Leica Application suite X and Rstudio version 4.2.1

For manuscripts utilizing custom algorithms or software that are central to the research but not yet described in published literature, software must be made available to editors and reviewers. We strongly encourage code deposition in a community repository (e.g. GitHub). See the Nature Portfolio guidelines for submitting code & software for further information.

Data

Policy information about availability of data

All manuscripts must include a data availability statement. This statement should provide the following information, where applicable:

- Accession codes, unique identifiers, or web links for publicly available datasets
- A description of any restrictions on data availability
- For clinical datasets or third party data, please ensure that the statement adheres to our policy

The datasets generated during and/or analysed during the current study are all available within the source data files provided with the manuscript. The RNA sequencing data that supports the findings of this paper have been deposited in the GSA database under accession code CRA015560. The molecular signature databases used can be found on the GSEAMSigDB website (https://www.gsea-msigdb.org/gsea/msigdb/index.jsp).

Human research participants

Policy information about studies involving human research participants and Sex and Gender in Research.

Reporting on sex and gender	N/A
Population characteristics	N/A
Recruitment	N/A
Ethics oversight	N/A

Note that full information on the approval of the study protocol must also be provided in the manuscript.

Field-specific reporting

Please select the one below that is the best fit for your research. If you are not sure, read the appropriate sections before making your selection.

- [x] Life sciences
- [ ] Behavioural & social sciences
- [ ] Ecological, evolutionary & environmental sciences

For a reference copy of the document with all sections, see nature.com/documents/nr-reporting-summary-flat.pdf

Life sciences study design

All studies must disclose on these points even when the disclosure is negative.

Sample size	For all experiments, a minimum n of 3, with all experiments performed twice was chosen. This was determined based upon similar studies in this field, as being sufficient for quantification purposes, and upon variability observed in past experiments of similar nature. In addition, this was chosen for practical purposes, taking into consideration the age of the mice required for most experiments (1 year) and animal costs.
Data exclusions	No data were excluded from the study
Replication	Mouse experiments were performed in biological duplicate or triplicate with at least three mice per group. Cell culture experiments were collected from three or four independent cultures for each sample. For all experiments, all attempts at replication were successful yielding similar results.
Randomization	Mice were fully randomized to treatments throughout the study.
Blinding	Experimental design and execution of in vivo and in vitro experiments were not blinded due to lack of trained personnel in the lab but all data analysis and image quantification were blinded.

Reporting for specific materials, systems and methods

We require information from authors about some types of materials, experimental systems and methods used in many studies. Here, indicate whether each material, system or method listed is relevant to your study. If you are not sure if a list item applies to your research, read the appropriate section before selecting a response.

Materials & experimental systems

n/a	Involved in the study	
☐	☒	Antibodies
☒	☐	Eukaryotic cell lines
☒	☐	Palaeontology and archaeology
☐	☒	Animals and other organisms
☒	☐	Clinical data
☒	☐	Dual use research of concern

Methods

n/a	Involved in the study	
☒	☐	ChIP-seq
☐	☒	Flow cytometry
☒	☐	MRI-based neuroimaging

Antibodies

Antibodies used	Antibodies used in this study are as follows:<br>total Pdgfrb: (4564S Cell Signaling, lot 6)<br>total Stat1: (Millipore 06-501, lot 3932871)<br>phosphorylated Stat1: (9167S Cell Signaling, lot 29)

B-tubulin: (15115S Cell signaling lot 3)
Gapdh: (2118 Cell Signaling lot 14)
phosphorylated Pdgfrb: (Y1009) (3124S Cell Signaling, lot 3)
phosphorylated P38: (4511S Cell Signaling, lot 13)
goat anti-perilipin: (abcam: ab61682, lot GR3456526-1)
mouse anti-alpha-smooth muscle actin: (NovusBio 2-34760V, lot 59-1XPABX170905)
rabbit anti-Ucp1: (abcam: ab10983, lot GR3432957-1)
mouse anti-DsRed: (Takara: 632392, lot 1906751A)
Cy5 donkey anti-goat: (Invitrogen A21447, lot 2465096)
Cy5 donkey anti-goat: (Jackson Laboratories 705-175-147)
Cy5 donkey anti-rabbit: (Invitrogen A10523, lot 2156245)
Cy5 donkey anti-rabbit: (Jackson Laboratories 711-175-152)
Cy3 donkey anti-rabbit: (Jackson Laboratories 711-165-152, lot 134989)
488 donkey anti-mouse: (Invitrogen A21042, lot 2160416)
488 donkey anti-mouse: (Jackson Laboratories 715-545-150)
ThermoFisher Scientific donkey anti-rabbit IgG (H+L) Cross-Adsorbed HRP 31458

Validation

All antibodies in this study were used and validated according to the provided data sheets and references for the specific technique (western blot, immunostaining, or flow cytometry) found directly on the manufacturer's website. All used dilutions can be found within the methods section of the manuscript.

Animals and other research organisms

Policy information about studies involving animals; ARRIVE guidelines recommended for reporting animal research, and Sex and Gender in Research

Laboratory animals

All animal experiments were performed according to procedures approved by the Cornell University Institutional Animal Care and Use Committee under the auspices of protocol number 2017-0063. Sma-CreERT2 mouse model was generously obtained from Drs. Pierre Chambon and Daniel Metzger 40. Sma-CreERT2 mice were combined with either Rosa26-tdtomato (stock #007914) or Rosa26-mTmG (stock #007676) mice from Jackson Laboratories. Sma-CreERT2 reporter mice were crossed with either PdgfrB D849V (stock #018435) or PdgfrBfl/fl (stock #010977) from Jackson Laboratories. Offspring were intercrossed for six generations prior to experimentation and were maintained on mixed C57BL6/J-129SV background. To induce recombination, denoted mice were administered one dose of TMX (50 mg/Kg; Cayman Chemical: 13258) dissolved in sunflower seed oil (Sigma, item no: S5007) for two consecutive days via intraperitoneal (IP) injection. After the final TMX injection, mice were maintained for seven days at room temperature prior to experimentation, as a TMX washout period. For cold temperature exposure, mice were housed in a 6.5ºC cold chamber (Power Scientific RIS70SD) or mice were maintained at RT (~23-25ºC; ~35% humidity). Mice were maintained in a vivarium on a 14:10-hour light/dark cycle with free access to food and water. All animal experiments were performed on 3 or more male mice per cohort and performed at least twice. All experiments were performed on male mice at 2-, 6-, 12-, and 24-months of age.

Wild animals

No wild animals were used in the study

Reporting on sex

Male mice were used in this study

Field-collected samples

No field collected samples were used in the study

Ethics oversight

Cornell University Institutional Animal Care and Use Committee under the auspices of protocol number 2017-0063.

Note that full information on the approval of the study protocol must also be provided in the manuscript.

Flow Cytometry

Plots

Confirm that:

☒ The axis labels state the marker and fluorochrome used (e.g. CD4-FITC).

☒ The axis scales are clearly visible. Include numbers along axes only for bottom left plot of group (a 'group' is an analysis of identical markers).

☒ All plots are contour plots with outliers or pseudocolor plots.

☒ A numerical value for number of cells or percentage (with statistics) is provided.

Methodology

Sample preparation

The iWAT SVF was isolated and resuspended in 1X PBS along with blue fluorescent reactive dye. Cells were then pelleted (1,200 rpm for 10 minutes), resuspended in 0.3-0.5 ml of FACS buffer (2.5% horse serum; 2 mM EDTA in 1X PBS with 1X protease/phosphatase inhibitor cocktail) and filtered through a 5 ml cell-strainer capped FACS tube (BD Falcon). Cell sorting was performed on BD Biosciences FACSAria Fusion or cells were analyzed on a Thermo-Fisher Attune NxT cytometry. Viable cells were gated from the blue fluorescent reactive dye negative population followed by singlet forward and side scatter pattern and mGFP+ viable cells were sorted. For recombination efficiency anaylsys, cells were stained for alpha-SMA (NovusBio 2-34760V) and analyzed for SMA/GFP double positivity. For phosphorylated Stat1 and total Pdgfr analysis, mGFP

	+ cells were fixed with 4% PFA for 1 hour at room temperature. Subsequently, cells were washed with 1X TBS and permeabilized for 30 minutes at room temperature with 0.3%TritonX-100 in 1X TBS. Cells were blocked with 5% donkey serum in 1X TBS for 30-minutes and incubated with primary antibody in 1X TBS with 5% donkey serum overnight at 4°C. Primary antibodies used were phosphorylated Stat1 (1:200; 9167S Cell Signaling) or total Pdgfr (1:200; 4564S Cell Signaling). After washing, secondary antibodies were applied for 2 hours at room temperature in the dark, then analyzed. Seconday antibodies used were Cy5 donkey anti-rabbit (1:200; Jackson ImmunoResearch).
Instrument	BD Biosciences FACSAria Fusion or cells were analyzed on a Thermo-Fisher Attune NxT cytometry
Software	BD FACSDiva Software version 9.4, Attune Cytometric Software 5.3.2415.0, and FlowJo software was used to analyze and collect data
Cell population abundance	For flow cytometry cell purity was determined using initial live/ dead cell staining and FSC/SSC gating to remove doublets. A minimum of 10,000 events was recorded for each experiment with >90% purity
Gating strategy	Viable cells were gated from the blue fluorescent reactive dye negative population followed by singlet forward and side scatter pattern. The preliminary FSC/SSC gates of the starting cell population were located at ~200K and ~75K, respectively. For antibody staining and applying the proper compensation matrix, a negative control and single stained sample was brought for each antibody and fluor used to specify the boundary between "positive" and negative" cell populations. No gating was applied for histograms.

☒ Tick this box to confirm that a figure exemplifying the gating strategy is provided in the Supplementary Information.
